# “The Dynamic Nature of Being a Person”: An Ethnographic Study of People Living With Dementia in Their Communities

**DOI:** 10.1093/geront/gnad022

**Published:** 2023-03-04

**Authors:** Linda Birt, Georgina Charlesworth, Esme Moniz-Cook, Phuong Leung, Paul Higgs, Martin Orrell, Fiona Poland

**Affiliations:** School Health Sciences, University of East Anglia, Norwich, UK; Department of Clinical, Educational and Health Psychology, University College London, London, UK; Faculty of Health Sciences, University of Hull, Hull, UK; Division of Psychiatry, University College London, London, UK; Division of Psychiatry, University College London, London, UK; Institute of Mental Health, University of Nottingham, Nottingham, UK; School Health Sciences, University of East Anglia, Norwich, UK

**Keywords:** Cognitive impairment, Ontology, Personhood, Social interaction, Social presence

## Abstract

**Background and Objectives:**

A dementia diagnosis can affect social interactions. This study aims to understand how people living with dementia act as social beings within everyday interactions in their local communities.

**Research Design and Methods:**

Focused ethnography informed by Spradley’s approach to data collection and analysis. Observations in community spaces.

**Results:**

Twenty-nine observations were undertaken in everyday social settings with 11 people with dementia who were part of a longitudinal interview study. Data consisted of 40 hr of observation, and researcher field notes. The overarching theme “*the dynamic nature of being a person*” encapsulates participants’ exhibited experiences in negotiating to attain and sustain an acknowledged place in their communities. Two subthemes characterized contexts and actions: (1) “Being me—not dementia”: Participants constructed narratives to assert their ontological presence in social settings. They and others used strategies to mediate cognitive changes evidencing dementia. (2) “Resisting or acquiescing to ‘being absent in place’”: Participants were often able to resist being absent to the gaze from others, but some social structures and behaviors led to a person being “in place,” yet not having their presence confirmed.

**Discussion and Implications:**

People living with dementia can actively draw on personal attributes, familiar rituals, objects, and social roles to continue to present themselves as social beings. Identifying how postdiagnosis people may self-manage cognitive changes to retain their presence as a person can help health and social care practitioners and families collaborate with the person living with dementia enabling them to have a continued social presence.

A diagnosis of dementia can negatively affect social relationships (Amano et al., 2021). It marks the outset of a journey of transitions, often seen by others as ending in the loss of the person: as lacking the essential elements for appearing to others as human. Families may talk of a process whereby the person living with dementia is “slipping away” (Feast et al., 2016). This absence of the person has been conceptualized as a “social death” in that the person has not lost their body but can lose their identity (Brannelly, 2011; Gilleard & Higgs, 2015; Sweeting & Gilhooly, 1997). Contrasting with this negative imaginary of loss and absence often bestowed by others are self-accounts which position the person’s experience as being hopeful and still active (Wolverson et al., 2010).

Importantly, living with dementia can be a fluid state shaped by social contexts and comorbidities; hence dichotomizing discourses are unhelpful to societal understanding (McParland et al., 2017). A diagnosis can mean living between and betwixt identities, as dominant medical and social discourses come to shape future identities (Birt et al., 2017). Diagnosis may confer “person with dementia” status, but their personal perception of self can encompass dominant constructs created before diagnosis, such as “spouse,” “entertainer,” or “craftsperson.” The key to living positively with dementia may lie in an individual retaining engagement in activities and with an identity as a person, beyond dementia (Birt et al., 2020; Wolverson et al., 2016). Although “self” is constructed internally by drawing on perceptions of who one is, it is maintained through interactions with others (Stets & Burke, 2003). The integrity of the “self” may be disrupted if the person is discounted by others (Beard, 2004; Sabat, 2002, 2008). Understanding how people with dementia may actively work to sustain continuity between their past and present selves can illuminate diverse discourses around the meaning of “realities” in dementia.

Commensurate with the personal experience of living with dementia are the theoretical discourses that guide care practices. Kitwood and Kontos provide two distinct approaches to care practice. Kitwood (1997) posited the idea of a “malignant social psychology,” which created an environment that undermined the personhood of the care receiver. He suggested that by understanding and supporting individual psychological needs rather than focusing on personal cognitive failures, their intrinsic personhood could be acknowledged and retained. Kontos’s work brings to the fore the idea that self-hood is a prereflective embodied state present even when people have advanced dementia, and that this embodied self may also be supported through relational care (Kontos, 2005). Over the past decade, the idea of personhood has also been integrated with the concept of social citizenship to recognize the social exchanges and “work” of those living with dementia (Bartlett & O’Conner, 2010). Although all three approaches have utility to practitioners in situating the challenges of cognitive impairment, there remains a need for a more fully developed understanding of how people with dementia enact everyday interactions in the community and how this might shape the “person” observed by others.

An individual’s experiences of “being” a person are central to our understanding of living with dementia. “Being” as a philosophical concept combines objective (exists as an independent being) and subjective (recognized as existing) aspects of reality. Smith (2019) locates “doing and being” actions within an individual’s agentic activity and within socially constructed domains. He argues actions are owned and intended, and so “embody meaning” constituting “the most fundamental manifestation of ‘personhood …’” locating “persons in a world of objects and, most importantly, others” (Smith, 2019, p. 2). Recognizing the interplay between social imaginaries and the person’s ontological presence (i.e., what is the nature of their existence) may bridge the divide between potentially dichotomizing discourses of social/physical and person/object.

Acknowledging ontologies that emerge here can equip us to explore the ontology (nature) of “being human” and how this may shift when living with dementia. People with dementia may distinctively characterize events and social interactions in ways shaped by their past knowledge, traditions, language, and culture (Gadamer, 1975/1996). This paper draws on ethnographic data to detail observed events where the person’s exhibited experience is contextualized in previous constructs of self. We consider how and how far these are now supported in the newer context of “being a person living with dementia,” observing features of social activity that support, or restrict, the person’s sustained self to continue to be an agentic person.

## Research Design and Methods

This ethnographic observation study was embedded within a longitudinal interview study research which sought to examine social discourses on dementia from the perspectives of those living with dementia, family carers, and people not affected by dementia (The Promoting Independence in Dementia study). Observing social situations enabled examination of the activity (behavior) carried out by the person living with dementia in a particular space (location).

The research question was “In what ways do people living with mild to moderate dementia experience social interaction in community settings?”

### Focused Ethnography

Our research methods needed to equip us to scrutinize the dynamics of interactions between people in social spaces, acknowledging that experiences are grounded in actions. This means interpretations are always understood through exhibited experiences. Ethnographic methods provide contextualized empirical understandings of peoples’ practices and actions, observed as people related to objects in place (Hammersley & Atkinson, 2007). Focused ethnography methods use short, intensive, linked field visits (Knoblauch, 2005). This method is pragmatically, ethically, and methodologically appropriate here in not imposing an unduly heavy burden on people. The time elapsing between each data collection event gives time for researchers to reflect on how their professional and cultural understandings shape how they interact in the observational setting; to consider emergent and analytical judgments on meanings and consequences of fieldwork interactions.

### Ethical Considerations

Ethical approval was obtained from the NRES Committee East of England (ref. 15/EE1034). We informed others in private spaces that researchers were present but that their data would not be recorded. To understand social interactions, it required us to report others’ ways of acting toward participants; we present this data without specifying details, to ensure anonymity.

At the first observation, the participant’s capacity to give consent was reviewed by clarifying questions on the purpose of the research, any risks or benefits to them taking part, and what they thought taking part would involve. All participants could give informed consent and nominate a consultee to act in their best interest if their mental capacity declined during the study. At each following observation, written consent was taken, and during observations, consideration was given to ongoing consent to the researcher’s presence (Dewing, 2007).

### Recruitment and Sample

We provided people living with dementia taking part in the interview study Promoting Independence in Dementia study (Csipke et al., 2021) with information on the observation study if they met the sampling criteria of engaging in activities outside the home. People were within 24 months of diagnosis, and all were active in everyday life. Therefore, they were considered to have mild to moderate dementia. Purposive sampling was guided by geographical area to include urban and rural areas in England; living arrangements to include those living alone and living with relatives; gender; and age. Participants living with dementia were invited to take part in up to four observations across 8–12 months. Where appropriate family carers, or other supporters, were invited to also be part of the observation, but the focus of the observation remained on the person with dementia. Recruitment took place from January 2016 to January 2017. We planned an indicative sample size of 10–15 but the methodological aim was to sample cases where there was potential for detailed data collection. Recruitment stopped when iterative analysis of ongoing observations indicated similarities both in activity patterns of people attending and in ways of individuals managing their interactions.

### Data Collection

Ethnographic data consisted of field notes recorded by the participating researcher including responses to informal ethnographic questioning, researchers’ reflections, and longitudinal interviews recorded in the main study. This paper reports the observation data, but an example of the link between the interview data and observations is shown in Supplementary Material (Section 1). Spradley’s (1980) accounts of ethnographic observation were used to structure field notes (see Table 1), prioritizing the nature and process of interactions, such as who started a conversation, how participants responded, strategies used to actively manage and mitigate cognitive impairment, or any overt or covert stigmatizing behavior directed to the participant or their companions. During observations, ethnographic questioning was used to clarify the meaning and “usualness” of events being observed. Field notes were reviewed within 24 hr to add details and reflective researcher memos.

**Table 1.** Field Note Guide to Structure Observations (Spradley, 1980)

Dimension	Description
Space	The physical place or places
Actor	The person or people involved
Activity	A set of related acts people do
Object	The physical things that are present
Act	Single actions people do
Event	As set of related activities people carry out
Time	The sequencing which takes place over time
Goal	The things people are trying to accomplish
Feelings	The emotions felt and expressed

Observation sites were selected in consultation with participants, providing contexts for micro- and meso-level connections (see Table 2). Researcher [L. Birt] carried out most observations within a rural county, with researcher [P. Leuong] conducting two observations within a city. Data collection ended in May 2018.

**Table 2.** Characteristics of Participant Sample and Observation Sites

Pseudonym	Age	Time since diagnosis	Living arrangement	Locality	Sites of observations
Mike	65–69	5 months	Lives with wife	Village (urban)^a^	Supermarket, cognitive stimulation club, and exercise/dance group
Fred	85–89	15 months	Lives alone	Village (urban)	Lunch club, church and shopping at local store
Holly	65–69	24 months	Lives with husband	Town	Supermarket, tea and chat club, town visit with sister, and cognitive stimulation group
Harriet	75–79	15 months	Lives with husband and daughter	Town	Local shop, tea, and chat group
Hannah	80–84	13 months	Lives with husband	Town	Supermarket, lunch club, and dementia singing club
James	80–84	13 months	Lives with wife	City	Art gallery and garden center
Maureen	80–84	6 months	Lives alone	City	Writing group and shopping using public transport
Simon	80–85	24 months	Lives with wife	Village (rural)	Local store, supermarket, dementia group, and pub lunch
Emma	60–64	24 months	Lives with husband	Village (rural)	Supermarket
Jack	80–84	4 months	Lives with wife	City	Supermarket, tea and chat, and garden center
Martin	80–84	30 months	Lives with wife	Town	Supermarket, day center, and dementia group

^a^Urban denotes a village with amenities such as shop, village hall, public building such as school or doctor’s surgery and with regular public transport. Rural villages were more isolated.

### Data Analysis

We treated social situations as an observable way to access culturally relevant actions, artifacts, and practices that people “*have learned or created*” (Spradley, 1980, p. 86). Drawing on Spradley’s accounts of domain analysis provided us with an auditable way of describing data. The first field notes were written to include researchers’ reflective memos. Then, each participant’s set of observations was repeatedly read, and the cover terms were noted. Cover terms are broad descriptors of actions and practices, for example, shopping, greeting others, and humor. The next step was within each cover term to further breakdown the actions, practice, and material things that took place generating “included terms.” Our particular focus was on if and in what ways people living with dementia might shape their action and practices. For example, the cover term, shopping, had the included terms of making a list, getting the trolley, etc. The next step in domain analysis was to consider the semantic relationship between the included term and the cover term. A semantic relationship provides a meaningful context, for example, getting the trolley is part of shopping, or a list is used for shopping (see Supplementary Material, Section 2).

Having organized and descriptively defined the experience of social interactions for people living with dementia, we moved to interpret exhibited experiences to explore how, within these sociocultural domains, the participants were appearing to present themselves as being a person (with or without a dementia identity). Our interpretation was informed by our previous work in the Promoting Independence in Dementia Study and our practice ethos that people can live as social citizens with dementia. This data set enabled us to examine if narratives of an agentic self, which were presented in interviews, were enacted in practice.

### Trustworthiness

We undertook a number of activities to enhance trustworthiness (Lincoln & Guba, 1985). Credibility was supported by the analysis of multiple observational data events per participant and across participants. Furthermore, the congruence between interpretations made on observation data and the participants’ reality could be explored by triangulating interpretations with interview themes. To ensure dependability that others would see the same things in the data, the research team met frequently to critically discuss how professional and personal subjectivities might shape our interpretations. We shared data with public members of the research group who had lived with the experience of dementia. We presented raw and analyzed data in multidisciplinary meetings to draw on diverse professional specialties and interpretations. It was not possible to undertake member checking due to the study design leading to a time lapse which would make the recall of events challenging. By providing details of observation sites and activities we might increase the transferability of results. However, it is important to note that this was a sample of people living with mild to moderate dementia undertaken in an English setting, so transferability may be limited by these boundaries. Using a systematic approach to data recording and analysis increases the audibility and hence confirmability of results.

## Results

Eleven people living with mild to moderate dementia took part (Table 2). Their mean age was 75 years (range 60–87 years); five were women. Most lived at home with a spouse, with two living alone. The results are illustrated with extracts from the researchers’ field notes.

The overarching theme, “*The dynamic nature of being a person*,” encapsulates exhibited experiences of participants as they negotiated a place in their communities while living with dementia. We contextualized each participant’s actions not just as “a person with dementia” but also as “a person with roles, responsibilities, and social presence.” We identified their active work, and how through deploying objects and routines they were observed to substantiate their presence. Two sub-themes specified actions and discourses of “being a person” with extracts from observations used to contextualize interpretations. Supplementary Material (Section 1) provides further examples illustrating how observation data aligns with the larger interview data set.

1. “Being ‘me’ – not ‘dementia’”: Participants shared personal narratives from before their dementia diagnosis. This could be seen to enable the self to be presented in distinct ways. Strategies such as deploying actors and objects appeared to mediate the cognitive changes that would evidence dementia. Other people supported their presence as a person beyond dementia.
2. “Resisting or acquiescing to ‘being absent in place’”: Participants were often able to resist being absent from the gaze of others, but some social structures and behaviors led to a person being “in place,” yet not having their presence confirmed by others.

### Being “Me” – Not “Dementia”

All participants acknowledged the reality of living with a dementia diagnosis. Nonetheless, people drew on previous roles, objects, and humor which could be seen as positioning them as more than their dementia diagnosis. They used strategies to minimize the impact of their cognitive decline and others in their social networks were important in supporting their presence as a person beyond dementia.

#### Roles, objects, and routines scaffolding a narrative as a person outside of dementia

Participants used objects to create opportunities to foreground their skills. For example, during a needlepoint demonstration at a coffee club, Harriet led the researcher to the table displaying needlepoint samples. She explained, she had been a seamstress and only her failing eyesight had stopped her, describing a physical rather than cognitive change, as leading to her ceasing her hobby. A new hobby could also be built on the foundations of a previous identity: Martin now sat in his chair at home, making wooden kit ornaments, which supported his ongoing narrative as a builder “good with his hands.”

Locally, participants were observed as making connections between buildings and their social selves. Passing a village hall elicited talk about their place in the village table tennis team or their organizer role in the church coffee morning. When attending church with Fred, he used buildings and objects to recreate his previous role as church warden:

Fred and I walked around the back of the church where there was a more modern graveyard and a seat. He explained, pointing out objects, how he and his wife had played a large part in getting the graveyard extended when he was the churchwarden. (2nd observation, Fred)

Fred maintained a significant and visible role as a bellringer, welcoming people and distributing hymn books, explaining he found the church service ritual comforting having been part of his routine all his married life. All participants valued routine, and going to familiar places with familiar people, whether through maintaining physical activities such as dance classes or more intellectual activities like quiz nights. Participants explained and demonstrated the importance of others knowing and accepting that they had dementia; this appeared to enhance their confidence in these activities.

Routines and known ways of behaving could be seen to facilitate participants’ agency in sustaining presence and acknowledged roles in everyday social groups. Victoria attended a writing group where the regular routine and anticipated actions created a “safe” space to act as oneself, here sustaining her peer status as a practicing writer:

Victoria listened to conversations about others’ work and nodded her head when she agreed with others’ points of views, commenting that another person’s story was ­interesting. Now it was her turn to read out her work. She did this in a clear voice, describing the relationship between her brother and sister-in-law. Some positive feedback about her work followed. She nodded to indicate she agreed with others’ comments. (1st observation, Victoria)

Here, Victoria’s presence is as a writer, not a person with dementia. Exchanging comments and praise of her work situated and “objectively” affirmed these skills, as she had not informed the group of her dementia diagnosis.

#### Using humor to have a presence as a person

Humor and laughter can join people together, as part of the group, as in this “tea and chat” session when Jack creates “mischief”:

Spotting two men at another table eating different food to the cakes on display, Jack urges his friend on to see what they are eating. His friend returns saying he couldn’t see what the food was. Jack says he will go over on the pretence of asking how the other men are and see if he can find out what they are eating. This he does, leaning in to talk, again there is laughter. Jack comes back and reports to others at his table that it was warm sausage rolls. People at the table suggest Jack asks for one, which he duly does, in good humour and in a discreet friendly way, with the result that he and his friend both are given a sausage roll from the kitchen helpers. (2nd observation, Jack)

Here, we interpret the meaning of these actions as Jack distinctively and successfully presenting himself as a person of humor. His congenial manner emphasized being comfortable in the presence of others, and being able to lead conversations and make others to feel at ease. He may have cultivated these skills in his teaching career, demonstrating essential aspects of his self-characterization as remaining intact.

#### Minimize the impact of cognitive impairment during social interactions

Small actions showed participants using techniques to mediate challenges of declining cognitive impairment, such as when poor recall of names or facts could have led to socially-difficult interactions. They deployed various strategies when introducing the researcher. A few introduced the researcher by name or as “the researcher,” others simply as “my friend,” enabling the researcher to introduce themselves by name.

When meeting others, participants often used simple, generic greetings. Holly, a friendly, socially-open woman, would smile and exclaim effervescently “*how lovely to see you*,” when people stopped to talk. She rarely used a person’s name or asked about specific details of their life. Rather, she moved conversations on with her smiling replies of “*how interesting*” and “*how lovely*,” “*illustrating how body language and being present in the encounter were as important within interactions as conversational content*.”

Participants were observed as using varying strategies to manage social interactions so as not to appear “incompetent.” Emma explained that when out in a new place to avoid getting lost, “*I tend to look round the room to find a face that I know*, *rather than remember where the table is*.” When alone in a shop, Emma was observed to use overhead store signs to situate her progress around the store. Others used subtle diversion responses to avoid direct questions. Here, Holly was attending a cognitive stimulation group:

At the end of the numbers activity the organiser goes through the questions, selecting people to answer. He turns to look at Holly and says “Holly, do you know the answer?” She has eye contact with him and replies “yes just thinking” then someone in the room shouts out the answer and she smiles and says “yes that’s right.” (3rd observation, Holly)

We suggest that the stalling phrase “just thinking” supported Holly’s presentation of self, in the created pause another person responded, with which she agrees with that reply. Occasionally, a pause during an interaction led to others prompting or answering for the participant. Although a few participants accepted this, others actively resisted, often saying “*I was just about to say that*.”

#### Presence as a person of purpose supported by others

Many participants seemed intent on retaining a presence as a person of purpose even as activities contracted due to changing physical and cognitive health. Several enjoyed time outside walking alone or with their dogs; an activity considered safe if neighbors or shopkeepers knew of their diagnosis and could help them if lost. Sometimes change was not solely due to reduced cognitive function. Fred had to relinquish his driving license due to failing eyesight, and so can no longer deliver the village newspapers. His friends know of this change and at the lunch club Fred is eager to sort out an alternative:

Fred says to the researcher ‘*I need to speak to his [another person in the group] wife is she here today, I can’t deliver the paper because I can’t drive*’. At this point another lady has arrived and she says ‘*Don’t worry Fred we have sorted it out next month I will drive you around and you can show me where the papers need to be delivered*.’ He says ‘*I can do the ones on foot stil*l’ she replies ‘*Yes I know you can keep doing those ones.*’ (1st observation Fred).

Here, Fred was seen to assert his “expert knowledge” of the delivery round, and the other person reinforces this by saying “*you can show me …*,” so confirming Fred’s still-useful role in hand-delivering the papers. When past activities were no longer possible, other people could support participants to build new activities: some joining new clubs, others exercising in new ways, and for a few, making more decisions, often around food, and shopping.

A visit to a shop was observed with most participants and the support offered to help the participant actively take part differed. Where support was offered to positively include the participant it tended to take the form of signposting and providing choice.

Mike’s wife enabled him to be active in shopping using various techniques:

She indicates to Mike where they are going in several different ways. She says the item they are next looking for, she usually also says that is on the right, or we need to take a left. She moves her arm in the direction they need to move in, she goes ahead of the trolley and Mike follows. She does not attempt to touch the trolley and she does not direct by touching Mike. He is watching her movements and changes directions as she does. (1st observation Mike)

This theme revealed purposeful narratives and tactics as people drew on features of talk and routines. A meaningful interpretation of this is that talk and routines could be deployed to self-characterize or constitute the person’s ontological presence as relevant and distinct from or even counteracting their lived-with condition. The places, events, and routines were all well known to participants and others scaffolded the strategies that were seen to be deployed.

### Theme 2 Resisting or Acquiescing to “being absent in place”

Most of our observations suggested people living with dementia were actively working to draw on past skills, objects, and rituals to position and reposition themselves as “being a person.” In this theme, we report data and interpretations in which other people’s actions could potentially “other” the participants and the steps they took that had the effect of repositioning themselves in the narrative and place. Yet for some, the structure of the place meant this was not possible despite the participants observed efforts, whereas one participant seemed to choose to be absent.

#### Foregrounding expert status

Accepting and acting on their willingness to share a diagnosis of dementia enabled people to share experiences in ways that inferred their expert status. When Harriet attended a church “tea and chat” session (not a dementia-specified activity) she did not hide her diagnosis, having shared it with her friends and others in her everyday life. The field notes provide interpreted meaning to Harriet’s ontology of “being expert”:

I (The researcher) sit at a table with Harriet and three friends chatting and drinking tea. The Vicar approaches me and asks if I am a new member; I explain I’m here with Harriet. Harriet says that I am doing research with her because of her dementia. The vicar then speaks directly to me saying he is setting up a dementia café and do I have any ideas. Immediately Harriet speaks saying she goes to a memory café. The vicar turns his attention to her and Harriet says *‘I have something’.* She rummages in her handbag producing an A5 sheet about a memory club she attends. She hands it to him and answers his questions reinforcing the importance of the word ‘memory’ rather than dementia. (2nd observation Harriet)

By focusing his attention on the researcher, the vicar is “absenting” Harriet. However, she agentically seeks and gains presence through articulating her expert personal experiences, becoming acknowledged and valued by others. All participants displayed awareness of their own presence and individuality, whether through their hobbies, their continuing social roles, or by holding on to aspects of their individuality, such as a love of fashion or being a host.

#### Countering others’ assertion of their having memory difficulties

Minor interactions between spouses illustrated participants’ agency in retaining control in everyday decisions. When preparing to go shopping, as Jack put on his shoes, his wife shouted to take the shopping bags, to which he replied “*don’t fore-think for me*, *I am just going to get them*.” Similarly, Simon’s wife possibly well meaningly put the newspaper voucher in his shirt pocket saying “*here don’t forget this*,” but he removed it, stating he wanted it in his wallet. Although many such interactions took place in private between couples, they also occurred within public spaces, here at a cognitive stimulation club:

Holly’s husband sat a few chairs away, not engaging in conversation with her until he looked across and announced loudly ‘*you have different earrings in’*. Holly reached her hands to her ears and her smile dropped then she replied ‘*oh yes well you know I like to be different’* she turned to the researcher and said ‘*I think it’s good to be an individual.*’ (Field notes 2nd observation Holly)

Holly was seen to take active steps to counter a comment potentially positioning her as “incompetent,” by refocusing it as displaying her marked individuality.

#### Being “absented” by others

In a few observation reports, the participant was present in the setting yet apparently absent from others’ gaze; this could be meaningfully interpreted as being positioned as less than a person by others’ actions. Often, these social interactions lacked those routines and objects that participants drew on elsewhere to construct their narrative and sense of security.

For example, when shopping with John the shop assistant directed questions to the researcher even though Jack was the person doing the activity and the researcher was slightly away from the till. On another occasion, John and his wife were having a pub meal with another couple where the man was living with dementia. The researcher noted the wives spoke openly about the challenges they faced caring for their husbands, while in the husbands’ presence and without acknowledgment of them.

Being socially “invisible” while physically present was also evident for James and his wife Sarah having lunch in a public café:

Towards the end of the meal two people walk behind myself and James, but Sarah can see them. As they leave the café she said to James that was xx and xx, they didn’t stop to talk. (1st observation James)

Sarah explained she noticed previous friends and acquaintances appearing increasingly to ignore them, no longer inviting them to events. This suggests that relatives of people living with dementia may also experience being “absented.”

When Martin attended a dementia-specific café, volunteers’ actions facilitated his narrative as a builder and contributed to conversations and music activities. However, in an unfamiliar place, a day center, the layout of the space and structure of the day constrained Martin’s “visibility.” There was a limited engagement between attendees or attendees and staff; Martin sat in silence, other than commenting to the researcher that he liked the music playing, appearing absent to others’ gaze, but nonetheless variously resisting this and continuously engaging in events in the room:

More time passes with no activity … Martin taps his foot in time to the music. Other attendees are dropping off to sleep. A lady attendee gets up and opens the window ­saying it is hot in here Martin agrees speaking across the room to her. No further conversation follows. A member of staff walks into the circle and says there is going to be a cooking activity; Martin turns to me to tell me his mother and his wife were both good bakers … all attendees remain in their chairs and watch a staff member show them how to make a cake with the promise of opportunity to ice them after lunch. During the demonstration Martin is closely watching and listening to the person and he mutters to himself “now add the eggs.” (2nd observation Martin)

In this setting, although Martin appears personally engaged, he is not having a presence due to the absence of scaffolding from others. Yet still, he could be seen as working to have a presence by attempting responses to others and initiating conversation with the researcher. In all observations, the characteristics of the place and types of activity were germane in enabling participants to direct their narratives and actions in ways that could confer personal presence.

#### Acquiescing to “being absent in place”

In our study, one participant stated they chose not to engage extensively with others, but to keep themselves distant. Hannah attended a day center, choosing to sit by the back wall. During observations, she independently entered the day room with clusters of tables for different activities, walking directly to a seat at the far wall facing the doorway. Hannah explained, she liked sitting there to see what was going on and who was coming in, but physically away from others. Occasionally, others’ actions drew her back into the group as and when another attendee encouraged Hannah to join their card game, which she did. Then during the “singing for dementia” session, Hannah was given a song sheet and asked to join in, which she did. However, on another occasion, she appeared to be absent when a visitor with a baby toured all tables but bypassing Hannah.

The theme examined ways people could actively resist or mitigate being absent in places, gain positive affirmations of their presence in interactions, or assert aspects of choice even when apparently acquiescing to fit into settings or groups.

## Discussion

This study adds to growing knowledge about the active way in which some people living with dementia draw on personal attributes and deploy novel expert knowledge to gain and sustain their presence as being a person in social settings; in part, this may be in response to the societal fear of being diminished by dementia. Our findings provide interpretative insights into the observed way people living with mild to moderate dementia created narratives, using nonverbal, as well as verbal strategies to position themselves as “persons beyond dementia.” This aligns with the review of Wolverson et al. (2016) which noted how people might transcend dementia and seek ways to maintain their identity. However, differing social structures, spaces and others’ responsiveness (i.e., othering; Doyle & Rubinstein, 2014) affected participants’ success in presenting themselves as a distinctive person “beyond dementia.” Importantly in discussing these results we acknowledge observations are from a bounded data set of people living with mild to moderate dementia in social settings which were generally familiar and nonthreatening to them. Many of the participants were physically fit and experiencing mild to moderate cognitive impairment. They might be culturally situated within the construct of the third age being relatively free of responsibilities and able to explore new opportunities. A few people with more advanced symptoms were seen to be increasingly reliant on others for access to communities and scaffolding their interactions. Yet, they were still able to share narratives as people with the agency even as their social worlds were shrinking and at a time when they or others may be fearing the terror of decline into a shadow self in the fourth age (Higgs & Gilleard, 2016). Importantly, in an observation study, determinism and meaning in a participant’s actions can only be inferred, but the contextual data on settings, actors, and explanatory questioning adds credibility to researcher interpretations.

We found people living with dementia presented purposeful narratives to create a “lasting legacy” of attributes established from their earlier roles and activities, supported by objects or routines, to position themselves as more than just “a person with dementia.” Continuing to represent predementia identities echoes. Weinreich’s (1986) suggestion that there is continuity between how a person dynamically construes their own identity in the present, how one presented oneself in the past, and how one wishes to be in the future. Retaining, reinforcing, and foregrounding elements of previous selves such as seamstress, builder, or writer can be considered to enable participants to constitute their ontological presence as distinct from identifying their selves as coterminous with just their condition—“a person with dementia.” When previous roles and activities had changed, connections to communities and materials such as the church, garden, or the village hall’s bricks and mortar, helped secure their lasting legacy through other means, substantiating their continuing social presence. Connecting with such visible physical objects might also make it harder for others to dismiss the person with dementia: to position them as without a past, present, or potential future existence, so as without ontological presence. Noting how people positioned themselves as “being a person who …” resonates with Hennelly’s review (Hennelly et al., 2021), identifying occupational and social roles as important in enabling people living with dementia to maintain their personhood. Importantly, such strategies were more successful when others recognized, acknowledged, and built on the person’s own story (Toubøl et al., 2020).

Our first theme highlights the work undertaken by the person living with dementia and others to enable them to be present in the social world. The work of gaining access to and membership in social spaces resonates with another community ethnography which indicated work is both personal and supported by others (Bartlett, 2022). We observed people actively working to counter differences’ caused by dementia, using humor and masking memory problems in social interactions. People often used nuanced skills such as deflection, avoiding using names in introductions, and using holding phrases such as “*how lovely to see you*.” This finding resonates with previous “impression management” to maintain an “acceptable self” (Beard et al., 2009; Birt et al., 2020; Caddell & Clare, 2010).

The second theme illustrates the agentic actions of people to draw on their dementia diagnosis to start to recreate new identities, especially in contexts where people might “other” them. Although accepting that some people struggle to accept a diagnosis, this finding illustrates that others may move to a postdiagnosis state of living as an active citizens with dementia (Birt et al., 2017). Although our older sample of people were not social activists (Bartlett, 2014), there were examples of people positioning themselves as experts because of their dementia diagnosis. This may be a way of resisting being absent. Nonetheless, there were examples of being absent by others and on occasions, this happens in a cared environment.

It appears vital to also acknowledge “choosing to be distant” as having implications for an agency in people living with dementia. Withdrawal from social interaction may be a consequence of the disease process (Porcelli et al., 2019), but it may also be perceived as a choice even in people with more advanced dementia (Ciofi et al., 2022). It appeared here that some people actively choose not to join in with group activities: physically distancing themselves from surrounding social activity, describing making a choice on when to “join in.” Without more closely investigating the intent of a person with dementia, we may ascribe their apparently more limited actions to apathy; a dementia symptom rather than their active choice. Avoiding social interactions can be a personal characteristic so a dementia diagnosis may not change persistent features related to “being a person” where people are less socially expansive throughout their lives. Reflecting on reasons and practices through which people may appear withdrawn might help actively support social interaction within their boundaries, rather than unthinkingly absenting them from opportunities to meaningfully social interact, or forcing an engagement.

Our ethnographic findings help conceptualize which physical places potentially enable people to construct social “presence.” There is evidence that different environments enable different types of community engagement for the person living with dementia (Gan et al., 2022). We found supermarket bustle often restricted chances for people to discuss and make decisions, whereas engaging with another person offering them quiet attention facilitated their narratives as “a person of account.” Future work may explore the psychosocial components of “place” and how these supports are being present as a person.

### Reflections on Methodology

Our results need to be considered within the constraints of the methodology. A common critique of naturalistic ethnographic research is that the researcher’s presence changes the nature of the existing activity as others see the researcher as “new” (Hammersley & Atkinson, 2007). Such critiques assume some pre-existing constant state, which many qualitative research approaches contest, given a dynamically changing world. This researcher’s adoption of a self-observing active role in the world of the person living with dementia supported an active interpretative analysis (Ingold, 2014). The presence of others, for example, support workers, family, general public enabled the researcher while observing, to fit within that activity and not to assume that the observed activity represented or disrupted some previous static state. Extended observations and repeated visits helped researchers formulate, and test accounts of patterns and routines constructed within each setting.

An important limitation is the sample’s lack of representation from ethnic minority communities. This sample where drawn for a larger sample where ethnic diversity was limited. There remains a need for further research as different cultural understandings of dementia could affect the ways in which a person may be enabled to perceive themselves, and be perceived and valued by others (Hillman & Latimer, 2017). We did, however, include people living alone as well as those supported by a spouse. When considering the transferability of results, it needs to be acknowledged this was a purposive sample of people with mild to moderate dementia who were able to engage with others outside of the home either with or without the support of others. Therefore, they had the physical and cognitive capacity to present as a person beyond dementia. Nonetheless, other research has suggested that people living with advanced dementia also enact and draw on previous roles to present themselves (Godwin & Poland, 2015). The experiences of being in the community for this sample were generally supportive and participants were accepting of their diagnosis. This is not the lived reality of all people living with dementia and people may make move through many stages as they adapt to a diagnosis (Birt et al., 2017; Hennelly et al., 2021).

A conundrum of any dementia research especially observations is that not all action and talk is a consequence of the diagnosis rather it is likely to be constituted through the person’s history, culture, belief, and further constructed within the research event. Furthermore, in an ethnographic method, the intent behind actions and the way an event is experienced, can never be fully known but can be meaningfully interpreted by the researcher. Therefore, it is important to acknowledge these interpretations are from the perspectives of researchers who although experienced in working with people living with dementia do not themselves have dementia. Taking findings back to people living with dementia may have led to more nuanced insights. Within this study, this activity was not planned so credibility in results was supported by the triangulation of this observation data to other data from the longitudinal study.

### Implications for Practice

Our results add to the understanding of ways in which people living with dementia may undertake actions to maintain their personhood presenting as a person beyond dementia. They often drew on past routines, roles, and hobbies even if they no longer necessarily fulfilled these. Considering Kitwood’s model of person-centered care, the identity of a person living with dementia requires a “*sense of continuity with the pas*t” and “*a story to present to others*” (Kitwood, 1997, p. 43). We found that opportunities to share that story could be scaffolded or “closed down” by others. The use of objects may be a scaffolding practice that can sustain communal social structures (Cicourel, 2013). Therefore, in practice, it may be helpful for practitioners to make time to know something about the person’s past. If dementia symptoms are more advanced this knowledge may need to be gained from family or close friends.

Kitwood’s concept has been critiqued as overlooking body, time, or dynamic agency in space, place, and interaction (Dewing, 2008). Our ethnographic findings appreciate these latter aspects as being essential to realizing being present as a human rather than a shadow self in social interactions. The observational evidence of people living with dementia engaging, managing, and presenting their selves as “person in place,” through interaction or actively withdrawing from others. There are psychosocial implications that facilitating opportunities for everyday social participation with others may support self-identity. Knowing the ways in which people may be supported to retain a subjective sense of self intact and with purpose is an important understanding for practitioners as discontinuities of self may lead to poorer psychological well-being (Clare et al., 2020).

## Conclusion

This ethnographic study of everyday social connections of people living with mild to moderate dementia has highlighted how they may be observed to work to construct and act on narratives of “persons beyond dementia.” Representing earlier-life roles, objects, and routines enabled people living with dementia to sustain and enact their presence in place and to present current and future narratives which extend their presented selves beyond that of a “person with dementia.” Others’ actions can importantly scaffold the person’s work by acknowledging and enabling the dynamic ontological presence of the person living with dementia. People with mild to moderate dementia may retain social status continuing to be recognized and affirmed within their communities.

## Supplementary Material

gnad022_suppl_Supplementary_Material

## Data Availability

The data that support the findings of this study are available on request from the corresponding author (L. Birt). The data are not publicly available due to their containing information that could compromise the privacy of research participants.

## References

[CIT0001] Amano, T., Reynolds, A., Scher, C., & Jia, Y. (2021). The effect of receiving a diagnosis of Alzheimer’s disease and related dementias on social relationships of older adults. Dementia Geriatric and Cognitive Disorders, 50, 401–406. doi:10.1159/000519581 34649243

[CIT0002] Bartlett, R. (2014). Citizenship in action: The lived experiences of citizens with dementia who campaign for social change. Disability & Society, 29, 1291–1304. doi:10.1080/09687599.2014.924905

[CIT0003] Bartlett, R. (2022). Inclusive (social) citizenship and persons with dementia. Disability & Society, 37(7), 1129–1145. doi:10.1080/09687599.2021.1877115

[CIT0004] Bartlett, R. & O’Conner, D. (2010). Broadening the dementia debate: Towards social citizenship. Policy Press.

[CIT0005] Beard, R. L. (2004). In their voices: Identity preservation and the experiences of Alzheimer’s disease. Journal of Ageing Studies, 18(4), 415–428. doi:10.1016/j.jaging.2004.06.005

[CIT0006] Beard, R. L., Knauss, J., & Moyer, D. (2009). Managing disability and enjoying life: How we reframe dementia through personal narratives. Journal of Aging Studies, 23, 227–235. doi:10.1016/j.jaging.2008.01.002

[CIT0007] Birt, L., Griffiths, R., Charlesworth, G., Higgs, P., Orrell, M., Leung, P., & Poland, F. (2020). Maintaining social connections in dementia: A qualitative synthesis. Qualitative Health Research, 30(1), 23–42. doi:10.1177/1049732319874782 31550999

[CIT0008] Birt, L., Poland, F., Csipke, E., & Charlesworth, G. (2017). Shifting dementia discourses from deficit to active citizenship. Sociology Health Illness, 39, 199–211. doi:10.1111/1467-9566.12530 28177147

[CIT0009] Brannelly, T. (2011). Sustaining citizenship: People with dementia and the phenomenon of social death. Nursing Ethics, 18(5), 662–671. doi:10.1177/0969733011408049 21893577

[CIT0010] Caddell, L. S., & Clare, L. (2010). The impact of dementia on self and identity: A systematic review. Clinical Psychology Review, 30(1), 113–126. doi:10.1016/j.cpr.2009.10.003 19896760

[CIT0011] Cicourel, A. (2013). Origin and demise of socio-cultural presentations of self from birth to death: Caregiver “scaffolding” practices necessary for guiding and sustaining communal social structure throughout the life cycle. Sociology, 47(1), 51–73. doi:10.1177/0038038512456779

[CIT0012] Ciofi, J. M., Kemp, C. L., & Bender, A. A. (2022). Assisted living residents with dementia: Being out in the world and negotiating connections. Gerontologist, 62(2), 200–211. doi:10.1093/geront/gnab113 34370003 PMC8827327

[CIT0013] Clare, L., Martyr, A., Morris, R. G., & Tippett, L. J. (2020). Discontinuity in the subjective experience of self among people with mild-to-moderate dementia is associated with poorer psychological health: Findings from the IDEAL cohort. Journal of Alzheimer’s Disease, 77(1), 127–138. doi:10.3233/jad-200407 PMC7592652 32804138

[CIT0014] Csipke, E., Shafayat, A., Sprange, K., Bradshaw, L., Montgomery, A. A., Ogollah, R., Moniz-Cook, E., & Orrell, M. (2021). Promoting Independence in Dementia (PRIDE): A feasibility randomized controlled trial. Clinical Interventions in Aging, 16, 363–378. doi:10.2147/CIA.S281139 33664568 PMC7921631

[CIT0015] Dewing, J. (2007). Participatory research: A method for process consent with persons who have dementia. Dementia, 6(1), 11–25. doi:10.1177/1471301207075625

[CIT0016] Dewing, J. (2008). Personhood and dementia: Revisiting Tom Kitwood’s ideas. International Journal of Older People Nursing, 3, 3–13. doi:10.1111/j.1748-3743.2007.00103.x 20925884

[CIT0017] Doyle, P. J., & Rubinstein, R. L. (2014). Person-centered dementia care and the cultural matrix of othering. Gerontologist, 54(6), 952–963. doi:10.1093/geront/gnt081 23921807

[CIT0018] Feast, A., Orrell, M., Charlesworth, G., Melunsky, N., Poland, F., & Moniz-Cook, E. (2016). Behavioural and psychological symptoms in dementia and the challenges for family carers: Systematic review. British Journal of Psychiatry, 208(5), 429–434. doi:10.1192/bjp.bp.114.153684 PMC4853642 26989095

[CIT0019] Gadamer, H. (1975/1996). Truth and method (G. Barden & J. Cumming, Trans.). Seabury Press.

[CIT0020] Gan, D. R. Y., Chaudhury, H., Mann, J., & Wister, A. V. (2022). Dementia-friendly neighborhood and the built environment: A scoping review. Gerontologist, 62(6), e340–e356. doi:10.1093/geront/gnab019 33564829

[CIT0021] Gilleard, C., & Higgs, P. (2015). Social death and the moral identity of the fourth age. Contemporary Social Science, 10(3), 262–271. doi:10.1080/21582041.2015.1075328

[CIT0022] Godwin, B., & Poland, F. (2015). Bedlam or bliss? Recognising the emotional self-experience of people with moderate to advanced dementia in residential and nursing care. Quality in Ageing and Older Adults, 16(4), 235–248. doi:10.1108/qaoa-08-2015-0038

[CIT0023] Hammersley, M., & Atkinson, P. (2007). Ethnography: Principles in practice (3rd ed.). Routledge.

[CIT0024] Hennelly, N., Cooney, A., Houghton, C., & O’Shea, E. (2021). Personhood and dementia care: A qualitative evidence synthesis of the perspectives of people with dementia. Gerontologist, 61(3), e85–e100. doi:10.1093/geront/gnz159 31854441

[CIT0025] Higgs, P., & Gilleard, C. (2016). Personhood, identity and care in advanced old age. Policy Press.

[CIT0026] Hillman, A., & Latimer, J. (2017). Cultural representations of dementia. PLoS Medicine, 14(3), e1002274. doi:10.1371/journal.pmed.1002274 28350889 PMC5370114

[CIT0027] Ingold, T. (2014). That’s enough about ethnography! Journal of Ethnographic Theory, 4(1), 383–395. doi:10.14318/hau4.1.021

[CIT0028] Kitwood, T. (1997) Dementia reconsidered: The person comes first. Open University Press.

[CIT0029] Knoblauch, H. (2005). Focused ethnography. Forum Qualitative sozialforschung [Forum: qualitative social research], 6(3), 44. doi:10.17169/fqs-6.3.20

[CIT0030] Kontos, P. C. (2005). Embodied selfhood in Alzheimer’s disease: Rethinking person-centred care. Dementia, 4(4), 553–570. doi:10.1177/1471301205058311

[CIT0031] Lincoln, Y. S., & Guba, E. G. (1985). Naturalistic inquiry. Sage.

[CIT0032] McParland, P., Kelly, F., & Innes, A. (2017). Dichotomising dementia: Is there another way? Sociology of Health & Illness, 39(2), 258–269. doi:10.1111/1467-9566.12438 28177143

[CIT0033] Porcelli, S., Calabrò, M., Crisafulli, C., Politis, A., Liappas, I., Albani, D., Raimondi, I., Forloni, G., Benedetti, F., Papadimitriou, G. N., & Serretti, A. (2019). Alzheimer’s disease and neurotransmission gene variants: Focus on their effects on psychiatric comorbidities and inflammatory parameters. Neuropsychobiology, 78(2), 79–85. doi:10.1159/000497164 31096213

[CIT0034] Sabat, S. R. (2002). Surviving manifestations of selfhood in Alzheimer’s disease: A case study. Dementia, 14, 11–19. doi:10.1177/147130120200100101

[CIT0035] Sabat, S. R. (2008). A bio-psycho-social approach to dementia. In M. Downes & B. Bowers (Eds.), Excellence in dementia care: Research into practice (pp. 70–84). Open University Press.

[CIT0036] Smith, S. (2019). Doing and being: A metaphysic of persons from an ontology of action. In D. Larrivee (Ed.). Neuroethics in principle and praxis: Conceptual foundations (pp. 1–15). IntechOpen. doi:10.5772/intechopen.77614

[CIT0037] Spradley, J. P. (1980). Participant observation. Thomson Learning.

[CIT0038] Stets, J. E., & Burke, P. J. (2003). A sociological approach to self and identity. In M. R. Leary & J. P. Tangney (Eds.), Handbook of self and identity (pp. 128–152). The Guilford Press.

[CIT0039] Sweeting, H., & Gilhooly, M. (1997). Dementia and the phenomenon of social death. Sociology of Health & Illness, 19, 93–117. doi:10.1111/j.1467-9566.1997.tb00017.x

[CIT0040] Toubøl, A., Moestrup, L., Ryg, J., Thomsen, K., & Nielsen, D. S. (2020). “Even though I have dementia, I prefer that they are personable”: A qualitative focused ethnography study in a Danish general hospital setting. Global Qualitative Nursing Research, 7, 1–11. doi:10.1177/2333393619899388 PMC6958651 31976359

[CIT0041] Weinreich, P. (1986). The operationalisation of identity theory in racial and ethnic relations. In J. Rex & D. Mason (Eds.). Theories of race and ethnic relations (pp. 299–320). Cambridge University Press.

[CIT0042] Wolverson (Radbourne), E. L., Clarke, C., & Moniz-Cook, E. (2010). Remaining hopeful in early-stage dementia: A qualitative study. Aging and Mental Health, 14(4), 450–460. doi:10.1080/13607860903483110 20455121

[CIT0043] Wolverson, E. L., Clarke, C., & Moniz-Cook, E. (2016). Living positively with dementia: A systematic review and synthesis of the ­qualitative literature. Aging and Mental Health, 20(7), 676–699. doi:10.1080/13607863.2015.1052777 26078084

