# Supplementary material for: “The Dynamic Nature of Being a Person”: An Ethnographic Study of People Living With Dementia in Their Communities
Source: Gerontologist. 2023 Mar 4;63(8):1320–9. doi: 10.1093/geront/gnad022 (PMC10474587; doi:10.1093/geront/gnad022)
Supplement: gnad022_suppl_Supplementary_Material [file gnad022_suppl_supplementary_material.docx]

**Section 1. illustrative data from observations and interviews**

|  | **Observation data** | **Interview data** |
| --- | --- | --- |
| **Theme 1 Being ‘me’ – not dementia** | | |
| **Roles, objects, and routines supporting a narrative as person outside of dementia** | Walking along the pavement Simon stops and tells me about the camber and how as a civil engineer he told the builders there had to be a camber.  I discuss the stained glass windows in the hall and this generates Martin narrative about his family work as a builder He says ‘I am good with my hands’. | Being a coast watch volunteer I’m not on my own. I’ve got really good eyes, so I’m useful when I’m there  We have a thing in the village called, ’Village Care,’ which I rather instigated 20-odd years ago, and it’s fantastic. Lots of volunteers that will do anything. This is one lady here who is on that thing. I have done it in the past when I have been normal, and they do everything for anybody, for free.  I'm probably involved less but fortunately the group is only ten minutes walk away so it's not a problem. I do go out on the boats occasionally still but in a different capacity, just as a passenger, not as somebody in charge of a boat's crew but the Association does understand that I'm not able to get down as often as I could and the book that I'm writing also covers the development the scout movement and then treating that as an activity so I'm still involved and I attend all of the public AGMs and things like that are necessary to go to and I keep in touch with people that I've known for a long time. I enjoy studying very much, there's other things in life to do but the advantage with the book that I'm writing is that I'm a published author so I belong to the Society of Authors and they run various meetings and talks. |
| **Using humor to have a presence as a person** | During the conversation Harriet had eye contact there was laughter, smiles and all responses were spontaneous and appropriate. We did not say who I was. Harriet says as her friend moves away ‘*I enjoy doing this walk I get to meet people for a chat, I have lived here a long time* (nearly 30 years) *and you get to know other people with dogs’. 13039a*  Often during the session Holly is looking ahead as if she is looking out of the window opposite. However it become apparent that she is listening to the ongoing conversations when one of the men cracks a joke she laughs to herself and this happens several times during the session.  During the quiz the organiser realised the answer was Rolf Harris she said as an aside ‘oh I have just realised the answer, perhaps not the best thing’ Mike was the only one in the group to quietly chuckle indicating that he had worked out the answer and the way Rolf Harris status had changed. | Hope I never lose my laughter, my chuckle muscles  It’s friendship and people you can rely on, have a laugh with, even if you’re feeling down and you’re annoyed with something. You still end up laughing, which is the best way to tackle it, really.  People see me in the village and ask how I am. I’ll say, ‘Oh, I’m completely mad today!’ and they’ll laugh, because they know my situation |
| **Minimize the impact of cognitive impairment during social interactions** | Fred says a general hello and heads to the man standing alone on left. He again goes to introduce me saying ‘this is my friend’ and introducing the man as my friend, … We manage this social situation by introducing ourselves rather than relying on Fred to provide names.  As we started walking toward the church Fred said that he would be saying hello to lots of people but that he wouldn’t be able to recall their names. We agreed that if need be I would introduce myself just by name.  At the end the activity the organiser goes through the questions and he selects people to answer saying their name looking at them and saying do you know the answer to Holly. She has eye contact with him when she is directly asked and replies ‘*yes just thinking*’ then someone in the room says the answer out loud and she smiles and says yes. | Yes. It’s better now – it’s much better now, with U3A. My word! There’s some people… this is going to sound bad, but on this little estate, if we go to the community room for two hours, at least one hour is talk about aches and pains that drives me mad. But to go to U3A I’ve found people have long and intelligent conversations. The next discussing group is about fracking – that’s going to be interesting. We’ve had talks on Brexit – can’t remember what we did last time. And I’ve just come from a science chat all about statistics and how they affect our world, and health all over the world, size of family compared to life expectancy – that sort of thing. It’s really been very interesting and people with brains to talk to!  I tend to look round the room to find a face that I know, rather than remember where the table is that I’ve gone to.  If I can’t remember someone’s name I just don’t bother. I say, ‘Now then, how are you doing? I haven’t seen you for ages,’ and it’s as if I still know them, but I don’t know their names, not all of them, but most of them.  I try and speak as part of the people who are there, if it’s a subject that I know something, – I know they’re talking about a subject in here, so I try and stick with the subject I know in here, because I’m more likely to come out with the right word. |
| **Presence as a person or purpose supported by others** | Martin’s wife frequently directs a question to him which triggers him to speak about the grandchildren.  We approach some potted plants on display and James’s wife stops and he stops alongside her. She says how lovely the plants are and does he think they should have one for the patio. He replies ‘yes I think so’. She then asks which colour he likes he does not reply she reduces the choice to the red or pink and he says the pink. She picks plant up and say I will put it in your basket.  In the supermarket Jane walks alongside john still giving the verbal instructions ‘*we need to go left down this aisle to get the oats*’ once in the aisle she moves forward in front of the trolley. Jane explains to me that John does not like it if she goes off to fetch an item so they go around together John says ‘*yes that’s right’*.  Holly husband says that sometimes he has to remind her to put her lipstick on but that this was important for her  On the third hymn it was the collection I had observed the two bags on the chair the lady at the end of the pew went to take the communion material to the alter she then come back and quietly said to Fred “*we need to do the collection but I can do it*” he turned to me and said “*oh it seems I was supposed to do the collection but I forgot”*. This conversation was whispered and happened at the back of the church. There was no one obviously looking or concern in the congregation when the collection bags were delayed and the collection still took place during the third hymn  Mike’s wife explains that at the dinner table when seated with strangers she felt she was doing too much of the talking on his behalf so they told people about his dementia and then found that when these people sat with them most slowed down their conversation and gave him time to respond. | I do make decisions, yes. I am the head of the family. I have a Power of Attorney in favour of my daughter who can therefore operate my bank account. She pays my bills out of that, but she discusses everything she is going to do with me before she does it  it was because it was easier to have what I was having than to have to read through a menu and make a decision. Now, if we know we’re going out, for example we went out last week with my family and we knew there would be a lot of noise and banter and laughter and the menu in front of us, so we went through the menu, online, before we went, and now we are down to two or three choices. It would have been just too daunting, he just cannot do it.  My wife is alongside with a little reminder … or a nudge. She gets me through. But you can hold a conversation for hours without actually saying the name.  I play golf with the lads regularly. I told them that if I do forget something they would have to remind me and that I wasn’t doing it purposely. And they understand. My daughter bought one of these things you press in your pocket, counter like. Needless to say I lost it [*inaudible*], but the lads are good but I always say to them that if I say I’ve had six and I’ve had five, they were just to tell me and I wouldn’t be offended. I would have to think back – they understand.  Help from other people and being given time. Time is a big help. As long as I’m given more time then I can usually work things out for myself. |
| **Theme 2 Resisting or acquiescing to ‘being absent in place’** | | |
| **Foregrounding expert status** | Harriet makes vicar aware of her dementia diagnosis and advising on preferred terminology | When I was diagnosed ... I'd do everything that I could to assist any university projects that were going and there's a slight possibility they might ask me to give some lectures but that really depends on my own mental health, you know, it might stay as it is or it might deteriorate but that's just one of the options that they put to me  When it dawned on me that I could help other people with dementia. That gave me a sense of purpose and until I got that I was wondering. I would almost say worried. Yes, the things you’d heard about people with dementia. So the joy to me was I knew I’d got it and then that I could do something about it in that I was going to help other people with it, |
| **Countering others’ assertion of their having memory difficulties.** | Harriet introduced me to her friends as the researcher who was with her because of her memory. To the bigger group inside I was introduced by name who was visiting with Harriet today.  A few days before the observation was due to take place Holly’s husband phoned to say that she was not happy about me going and meeting ‘those’ friends, she was happy to be part of another observation. | The travelling to meet different people. It makes you feel normal again, because you’re travelling just as you used to. You might have had to put in lots of work to do it, but you’re still able to do it, you’re not stuck in the house.  l’m not daft, I’m just memory loss. I haven’t lost my brain altogether.  Explaining why important to be part of a quiz team: It makes me feel part of something again, because I had to give up work. I think I would have had to give up anyway, but I was made redundant, which is quite fortunate in the circumstances. So, it just means I’m part of something and with the quizzes in the village it’s a way to see the people locally. |
| **Being ‘absented’ by others** | Holly’s husband comes into the room, he see me and comes over directly to speak to me not introducing himself to his wife and she is unaware that he is here as she is looking straight ahead out of the window.  Simon makes comments to me about the transgender police force story wife says ‘*never mind that just sit and read that section’*. She leaves the room then Simon gets up to show me a picture of people hanging off a sky scrapper we chat about having a head for heights and I said he must like heights in mountain rescue he says ‘*not like that always had my feet on* rock’.  At the self-service till we waited for a minute before she came over and Jack said he needed the alcohol checking she replied in an abrupt tone ‘*you could have continued shopping’*; he did not respond to this comment as he was sorting through his bag for the newspaper. He then showed her the newspaper and voucher without speaking she took this from him and scanned them. She then looked towards me I was stood near the bagging area slightly behind Jack and she said directly to me ‘*did you use any bags’*; Jack replied no so she hit another button on the screen and left us to continue the shop. | I feel I could go to town on my own, however I’m not  allowed. J doesn’t want me getting lost  I am no longer allowed to babysit children I think my daughter is a bit frightened of what might happen and she’s trying to protect the children from me. Not that she thinks I’d harm them but she doesn’t want the children to make fun of me if I’ve done something. At the moment it’s not anywhere near there. She’s protecting me and the children. She’s looking after both of us, so that neither of us get hurt by anything.  I haven’t lost any friends – in fact, they are probably more in contact now than before. Work was different. Colleagues thought I was stupid – people that I worked with but didn’t know very well. That was hard because I really loved my job.  Harriet says to me that when they got her diagnosis she was made redundant the family said she had to stop cooking, cleaning, shopping and she feels she has no purpose. |
| **Acquiescing to ‘being absent in place’** | During the sermon Fred was sat back in the pew and his eyes were either down or closed. When the sermon finished he turned to me and said in a quiet voice “I didn’t hear any of that”. Afterwards when we were discussing the service he said he went it was a routine it was what he always did but he didn’t really follow the sermon or prayers (i.e all the parts which are spontaneous and changing every week).  He takes me over and introduces me to Hannah. She is sitting in a high backed chair at the edge of the room facing into the room, with a view of the door through to the room. I sit alongside W, she has her eyes open, often she sits at rest with her eyes closed. I start a conversation. We chat about the day centre and she says she likes coming here and she likes this seat as she can see who is coming and going. She does not pass any comments about who is arriving or the actions of any of the other people. | The things which I was doing normally and the rule coming back immediately, knowing which rule it was and what I should do, I was finding I had to wait – I felt for ages – before I could give a decision and I didn’t think that was fair on the participants. The Parish Chairman – I was Chairman and I had difficulty… well, not difficulty, I found following the meeting sometimes more difficult than it used to be. Nobody else noticed, none of the Councillors or anybody there, so when I said I’d better give it up they were very much surprised, but they understood why  Once when I thought I made a fool of myself and I gave up being Chairperson. I did that in too much of a hurry. But I’m fine, I really am  I try to be involved but I don’t want to be involved, if you know what I mean, with all these committees. Just let somebody else have a go. Try not to be tied what with the old memory and things like that – new faces and things like that. |

**Section 2. Domain analysis moving from a social situation to discovering a cultural domain, which is a category of cultural meaning.**

| **Included terms** | **Semantic relationship** | **Cover term** |
| --- | --- | --- |
| Supermarket  Village shop  Garden centre | Is a kind of | shop |

| **Included terms** | **Semantic relationship** | **Cover term** |
| --- | --- | --- |
| Making a list  Getting there  Choosing trolley  Talking to people you know  Pushing the trolley  Choosing food  Sharing decisions about what to buy  Checking the list  Comparing prices  Choosing till  unpacking trolley  packing bags  paying for items  stopping for coffee  leaving store  loading car  driving home  unloading car  putting items away  handing over money | Is a stage of | shopping |

| **Example from data** | **Included term** | **Semantic relationship** | **Cover term** |
| --- | --- | --- | --- |
| Martin  Looking at the list he said we would start with the veg these counters were directly in front of us. He walked towards the veg and looked up and down the set out counter before picking up a pre-wrapped broccoli head. H explained that he gets pre-wrapped as this is easier when he gets to the self-service counter. He then looked at carrots, picking one packet up and replacing, explaining they were two large then picking an organic packet up which he explained were a better size | Making a decision | Is a stage of | Shopping |
| Martin selected the start scanning button and then carefully took each item from his shopping bag scanned it and laid it gently on the goods receiving tray. The first item he had to turn it round to find the bar code when we got to the broccoli he turned to me, smiled and said ‘*see simple no searching for item’*.. Once all items were scanned H selected to pay by debit card. This was a contact less payment and we chatted about how this was new and he liked it but only up to 30 pound. | Paying for items | Is a stage of | Shopping |
| Simon  The newspaper rack is immediately on right and Simon reaches the telegraph glances at front page then takes it to the counter. He and shopkeeper say hello shopkeeper scans the paper and H hands over the small voucher which he has immediately located in his trouser pocket. He walks back to walker which is just inside the door and puts the paper in the bag on walker. He pauses and I wonder if he will remember the milk. He then says to himself ‘*yes milk*’ and walks past the papers to the fridge. …. | Making a decision | Is a stage of | Shopping |
| He goes towards the counter to pay. At the counter he gets a handful of coins out of his trouser pocket and places them on the counter the shop keeper says it is £2 and he helps H find a £2 coin. H says thank you and God bless you to the shopkeeper and the shopkeeper says have a good day as they talk they are pushing their knuckles together in the air together | Paying for an item | Is a stage of | Shopping |
| Fred  He pulls a scrap of paper out of his pocket on which he has written his list, tea, sugar and soap. In the shop Fred quickly finds the tea bags and chooses between two makes and two sizes then he says now where is the sugar I point it out below the tea and he picks this up then he asks the staff where is the soap and the lady says her husband has just gone to back of shop to show him. There is a choice of two and he chooses imperial leather the one which is in his bathroom | Making a decision | Is a stage of | Shopping |
| When Fred is paying he pulls a handful of mixed coins from his pocket and lays them on the post office counter saying to the lady ‘is there enough there, I don’t know where all these coins come from’. 13032a | Paying for items | Is a stage of | Shopping |
